# Retinal Contrast Transfer Functions in Adults with and without ADHD

**DOI:** 10.1371/journal.pone.0061728

**Published:** 2013-05-02

**Authors:** Emanuel Bubl, Michael Dörr, Alexandra Philipsen, Dieter Ebert, Michael Bach, Ludger Tebartz van Elst

**Affiliations:** 1 Department of Psychiatry and Psychotherapy, Albert-Ludwigs-University of Freiburg, Freiburg, Germany; 2 University Eye Hospital, Albert-Ludwigs-University of Freiburg, Freiburg, Germany; University of Regensburg, Germany

## Abstract

In previous studies, we found a strong reduction in contrast perception and retinal contrast gain in patients with major depression, which normalized after remission of depression. We also identified a possible role of the dopaminergic system in this effect, because visual contrast perception depends on dopaminergic neurotransmission. Dopamine is also known to play an important role in the pathogenesis of attention deficit hyperactivity disorder (ADHD). Therefore, in order to explore the specificity of retinal contrast gain as a marker of depression in comparison with other psychiatric diseases, we recorded the pattern electroretinogram (PERG) in patients with ADHD. Twenty patients diagnosed with ADHD and 20 matched healthy subjects were studied. Visual pattern electroretinograms were recorded from both eyes. The contrast gain of the patients with attention deficit disorder (ADD) did not differ from the control group, nor did the contrast gain of any ADHD subgroup (predominantly inattentive or combined patients). In the healthy subjects, a significant correlation between depression score and contrast gain was found. As the contrast gain in an earlier study clearly separated the patients with depression from the controls, we assume that retinal contrast gain might be a specific marker in depression.

## Introduction

Increasing evidence points to abnormalities in vision in depressive disorder [1]–[4]. In two recent studies, we found a significant reduction in contrast processing in patients with depressive disorder. In the first study, patients with major depression presented reduced psychophysical contrast sensitivity [5]. The following study documented electrophysiologically a very strong reduction in pattern electroretinogram-based contrast gain in patients with major depression [4]. The pattern electroretinogram (PERG) is an electrophysiological response recorded at the cornea in response to visual pattern stimulation [6]. It predominantly represents the activity of the retinal ganglion cells [7], [8] and, thus, can serve as an objective surrogate marker of retinal information processing from the photoreceptors to the beginning of the optic nerve. The PERG-based contrast gain discriminated patients with major depression from controls with a specificity of 92.5% and a sensitivity of 77.5%. In a previous study [9], we found that this abnormality in contrast gain normalized in the context of remission of depression, whereas it remained abnormal when patients did not remit from depression, despite antidepressive therapy.

Very similar findings in contrast sensitivity and retinal contrast processing have been reported in patients with Parkinson's disease (PD). In earlier studies, we found that contrast sensitivity is reduced in PD, whereas contrast adaptation is not affected [10]. This result points to the retina as an important site of dysfunction in PD. In a subsequent study, patients with PD displayed a significantly reduced retinal contrast response [11]. Thus, the empirical pattern of reduced retinal contrast processing (with the objective electrophysiological signal PERG) is found in PD and major depression, except that these abnormalities normalize in depressed patients following remission. Since PD is the paradigmatic disease of dopaminergic dysfunction and retinal contrast perception is modulated via dopaminergic amacrine cells [12]–[15], we hypothesized that our finding of reduced retinal contrast gain in major depression is linked to reversible states of systemic dopaminergic dysfunction in depressed patients [9].

Against this background, we assessed the specificity of reduced retinal contrast gain in depression with respect to other neuropsychiatric disorders with a link to dopaminergic dysfunction, namely, attention deficit hyperactivity disorder (ADHD).

ADHD is well known in child and adolescent psychiatry, and its lifelong persistence is making an increasing impact on psychiatry in general [16], [17]. As a pathophysiological mechanism, an alteration in the adrenergic and dopaminergic systems has been proposed [18]. Until now, little has been known about the visual system of adult patients with ADHD, but there are reports of reduced visual perceptual sensitivity in psychophysical testing with simultaneous recordings of event-related brain potentials in children with ADHD [19]. Furthermore, a reduction in contrast sensitivity has been found in children with ADHD [20], which has been suggested to be even more prominent in color perception [21]. Van der Stelt described deficits in discriminating discrete stimulus events in color selective attention tasks [22], and there have been reports of an altered VEP response variability in children with ADHD [23]. Moreover, in one study, children with ADHD presented reduced visual acuity, which normalized following treatment with methylphenidate [24]. Furthermore, we found evidence of volume loss in the primary visual cortex of adult ADHD patients [25]. Finally, reduced spatial inattention in ADHD children has been associated with an alteration in the dopamine transporter gene (DAT 1) [26].

Given the hypothesis that retinal contrast perception is modulated by the dopaminergic system, we chose ADHD as the first psychiatric condition in which we wanted to test the specificity of our findings of reduced retinal contrast gain in major depressive disorder. Thus, the aim of the present study was to investigate a possible dysfunction of visual contrast processing at the level of the retina in patients with ADHD by assessing their retinal contrast gain. Given that retinal contrast gain is reduced in patients with depression, the study tested the specificity of that finding.

## Materials and Methods

Patients were recruited at the Department of Psychiatry of the University Hospital of Freiburg. They gave written informed consent to participate in the study, which was approved by the ethics committee of the Albert-Ludwigs-Universität Freiburg. The participants met the DSM-IV criteria for attention deficit disorder (ADD), classified as either ADHD of the combined type or predominantly inattentive or hyperactive type (DSM-IV: 314.00-01). The exclusion criteria were the presence of any other DSM-IV first-axis psychiatric disorder, any other general neurological or medical condition, or any eye disease, except for correctable refractive errors. The ADHD diagnosis was assessed by senior consultant psychiatrists on the basis of a detailed psychiatric interview that integrated common psychiatric and somatic differential diagnoses and the patients' medical histories. In addition, since ADHD is not included in the SCID-I, the investigator rated 18 items corresponding to the current DSM-IV-criteria for ADHD adapted for the special needs for adults as proposed by the German Medical Association (ADHD Checklist) [27]. All patients were also assessed psychometrically using the Wender Utah Rating Scale and Conners' Adult ADHD Rating Scales (CAARS) [28]–[30].

Eleven patients fulfilled the criteria for ADHD of the combined type (DSM-IV: 314.01) and nine for the predominantly inattentive type (DSM-IV: 314.00). The control group consisted of 20 age- and gender-matched healthy subjects without a history of neurological or mental disorders, all of whom scored in the normal range on CAARS and the ADHD Checklist. All subjects had a visual acuity above 20/25 wearing appropriate correction at the distance used for visual stimulation [31].

### Stimulation

**Figure 1:**
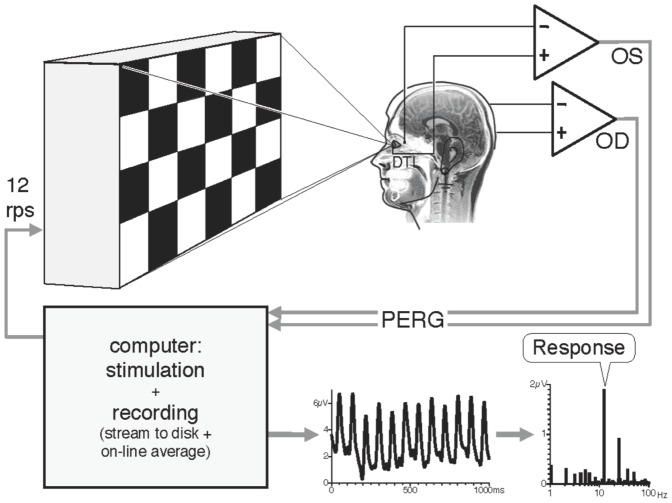
Schematic diagram of the recording setup.

Stimulation, recording, and analysis were performed by the EP2000 system [32]. In a dimly lit room, the stimuli were generated with a resolution of 800 × 600 pixels at a frame rate of 75 Hz and displayed on a raster-scan display, covering a field size of 32° × 27.0° at the observation distance of 57 cm with a mean stimulus luminance of 45 cd/m^2^. For illustration, see Figure 1.

To quantify the PERG-based contrast gain, a sequence of five checkerboard stimuli with 0.8° check size, contrast-reversing at 12 reversals per second, was presented with Michelson contrasts of 3.2%, 7.3%, 16.2%, 36%, and 80%. Each contrast level was presented for 10 s, and then the next contrast was applied, finally recycling to the first contrast level. This interleaved sequence was repeated until 80 artifact-free sweeps per contrast (1.0-s length each, containing 12 responses) were accumulated. The interleaved blocking ensured that any sequential effects (e.g., fatigue) were distributed equally across all contrast values. The sequence was repeated once, and further analysis was based on the vector average of each recording pair.

The PERG signals were recorded simultaneously from both eyes, using DTL electrodes placed at the lower limbus of each eye [33]. These were referenced to gold cup electrodes at the ipsilateral outer canthi; one earlobe was grounded. The subjects were requested to blink only infrequently during recording and to maintain a relaxed pose. Sweeps exceeding ±130 µV were rejected as artifacts. The subjects reported small digits appearing randomly every 20–30 s in place of the fixation cross displayed to facilitate and monitor correct fixation and accommodation.

The potentials were amplified, filtered (first order 0.5–100 Hz), and digitized at 1 kHz with 16-bit resolution. To prevent temporal aliasing, all timing (stimulation, analog sampling, sweep length) was related to the stimulus monitor frame rate [34].

**Figure 2:**
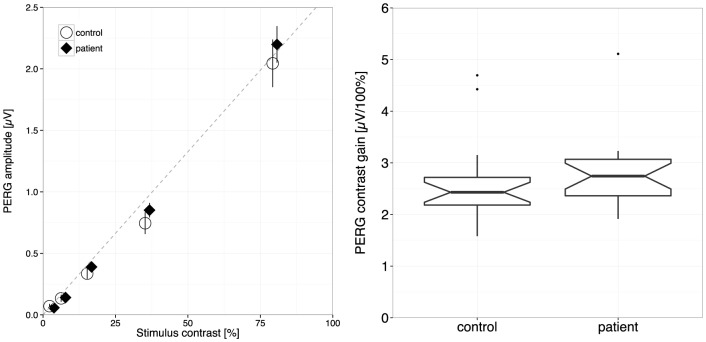
Comparison of contrast gain between patients and controls. **Left**. PERG magnitude versus contrast for patient and controls. The antennas represent ± SEM. The dotted line connects the origin and the mean of the amplitudes at 80% stimulus contrast. **Right**. Group PERG-contrast gain, grand average. Normal controls (left) and patients with ADD (right) do not differ significantly. [Box-plot details on the right: the median is indicated by the thick horizontal lines, the notches represent a 95% confidence interval for the medians, the box covers the 25–75% percentile range, the “antennas” indicate the range, and outliers are indicated by circles.].

Off-line, all traces were Fourier-analyzed to calculate the magnitude spectrum. From this, a noise-free response estimate was extracted [35], [36]. The second and third harmonics were combined via their quadratic mean (RMS value). A linear model of these spectral response magnitudes versus stimulus contrast yielded the contrast response function “PERG-based contrast gain,” quantified by the slope of the linear model (Figure 2 [4]. This slope will be termed “PERG-based contrast gain” or “contrast gain” throughout the paper. For a signal like the PERG, where the contrast transfer function is linear, the slope and contrast gain coincide.

The total duration of the recording was approximately one hour per subject.

### Data analysis

We tested for statistical significance on contrast gain and contrast and interaction using a 2-factorial ANOVA. Post-hoc analysis was performed to test the effects of type of diagnosis.

Correlations between contrast gain and the score on CAARS and the ADHD Checklist were calculated. Given our earlier reports of significant correlations between the extent of depression as measured with the Beck depression inventory (BDI) [37] and retinal contrast gain, we repeated these analyses for the control and patient groups in order to check the replicability of this earlier finding.

## Results

We measured the PERG-based contrast gain in 20 unmedicated patients with a current diagnosis of ADD (Table 1).

**Table 1:** Participant characteristics: gender, age, CAARS (ADHD Symptoms Total; DSM IV Inattentive) and contrast gain of the study group.

Group	Age	ADHD Symptoms Total	DSM IV Inattentive	Contrast gain
Patients (n = 10 male, 10 f.), mean±SEM	33.5±2.8	27.7±2.5	15.6±1.1	2.55±0.19
Healthy control ( = 10 male, 10 f.) mean±SEM	33.8±2.7	5.55±0.80	2.5±0.4	2.56±0.19

### Perg

Figure 2 (left) displays PERG amplitude vs. contrast per group, and (right) the contrast gain ( = slope) per group. The corresponding ANOVA results (amplitude versus group x contrast) revealed a highly significant effect of contrast (F = 1275, p<.0001), no effect of group (F = 2.1; p = .15), nor any interaction (F = 1.9; p = .17). The missing effect of group on contrast processing is also obvious from the overlap in the right panel (F = 0.59, p = .45).

### Contrast processing versus subdiagnosis

Eleven of our patients met the DSM-IV criteria for ADHD of the combined type and nine for the predominantly inattentive type. In the post-hoc comparison, no significant difference was seen (p>.1 in all groups).

### Artifacts

The number of artifacts is a quality estimate of patient cooperation. For example, when the participants are particularly restless, or execute frequent eye movements or blinks, the number of artifacts rises. The number of artifacts did not differ significantly between the two groups. The control group presented a mean 116 (SE = 92) sum of all recorded artifacts, compared to 102 (SE = 79) in the patient group (T = .504, p<.617).

### Contrast gain and the Beck depression inventory

**Figure 3:**
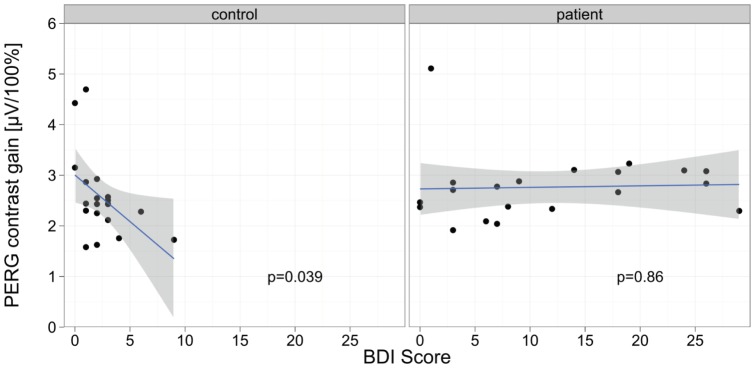
Correlation between PERG contrast gain and BDI for the two study groups. The p-values are indicated per group, the line represents a linear regression, the gray area indicates ±SEM of the regression line.

As in our earlier reports, we found a significant correlation between the BDI and retinal contrast in the control group (r = –.476; p = .039), despite the low variability of BDI ratings. We did not find such a relationship in the patient group. None of the patients had been clinically diagnosed with depression (Figure 3).

### Contrast gain and ADHD rating scales

There was no correlation between any CAARS rating scale (r = .096; p<.56) or the ADHD Checklist (r = −.014; p<.93) and contrast gain.

## Discussion

The results demonstrate that the pattern electroretinogram, which was reduced in patients with major depression in previous studies [4], [9], did not differ between the patients with ADHD and the controls. The patients with ADHD, in fact, presented a slightly steeper but non-significant mean contrast gain (Figure 2).

With respect to depression, we replicated our earlier finding of a significant correlation between BDI scores and contrast gain in the healthy control group (Figure 3). This is of note because the variance of BDI scores in our healthy control group was very low, that is, it only varied from 0 to 9 in the normal range of this depression instrument. In contrast, in the ADHD patients, there was no such correlation. Furthermore, in the ADHD group, there was no significant correlation between any ADHD rating scale and the retinal contrast gain.

Therefore, with this study, we found some specificity of retinal contrast gain as a marker of major depression, at least in comparison to ADHD in adulthood. Of course, specificity to other neuropsychiatric disorders, such as schizophrenia, schizoaffective, or bipolar disorder, needs to be examined as well, and a direct comparison between different disorders must be carried out.

We assumed that reduced retinal contrast gain in major depression reflects a systemic dopaminergic dysfunction, objectively measurable at the level of the retina. Thus, it might be surprising that retinal contrast gain was not altered in patients with ADHD, since retinal contrast is modulated by dopamine and dopamine plays an important role in ADHD [38]. The difference in the PERG signal illustrates that the pathophysiologies of major depression and ADHD are not identical, at least with regard to retinal dysfunction. This suggests that different pathophysiological mechanisms underlie ADHD and major depression. It is possible that other mechanisms of dopaminergic pathophysiology from the presynaptic to the synaptic, postsynaptic, or intracellular level are involved in the two entities.

Our primary goal was to test the specificity of retinal contrast gain abnormalities for major depression. Therefore, we used the identical paradigm as in our previous study. In this context, one has to consider that different stimulation and testing paradigms analyze different aspects of retinal function. Generally, contrast stimuli with high spatial frequency (small check sizes) are sensitive to changes in the D_2_-receptor family, rather than, for example, in the D_1_-receptor [39]. In major depression, an alteration in the D_2_-receptor family has been proposed [40]. On the other hand, a change in the D_1_-receptor family is discussed as one target element in ADHD [18]. Thus, coarse gratings with a lower spatial frequency (big check sizes) might be more sensitive to reflect visual alterations in ADHD than the spatial frequency used here. The chosen paradigm was able to detect abnormalities in patients with depression, but not in the ADHD group. Thus, patients with ADHD might have normal retinal contrast gain, or, alternatively, we did not choose the best stimulus paradigm, which could elicit differences in patients with ADHD. It might be preferable for further research into ADHD to explore lower spatial frequencies ≤ 0.3 cpd (corresponding to a check size of ≥ 2.4°), since it has been suggested that changes in the D_1_-receptor function are best detected there [12].

Some further limitations must be taken into account. Patients with ADHD obviously suffer from attention problems, and these might have hampered our results. Could it be that inattention led to a reduction in contrast gain that would have been detected otherwise or could have camouflaged reduced contrast gain in the ADHD group? In the present approach, we minimized a confounding effect of attention, since we used an electrophysiological task that is largely independent of attentional processes. The patients just viewed a computer screen displaying checkerboard patterns of different contrasts. We ensured that the participants fixated and focused by asking them to read out randomly presented digits from the screen center. The correct responses and artifacts did not differ between the two groups. In addition, the 12-Hz signal recorded from the retina illustrates that the subject looked at the grating, since no 12-Hz signal would have been produced otherwise (see Figure 1).

Another issue is the moderate sample size of 40 subjects (20 ADHD and 20 control subjects). A larger sample might have picked up a more subtle difference in signal; thus, our result could be a false negative due to an underpowered sample. However, the size of this sample relates well to that of other similar pilot studies, and on the basis of our earlier study of patients with depression, we know that such a sample can produce very strong and clear difference signals [4].

None of the patients in the ADHD group had a clinical diagnosis of depression; nevertheless, some of our patients presented high BDI ratings. In this case, the BDI is not a good measure for the severity of depression [41]. The false positive BDI scores might be explained by an overlap in ADHD symptoms with questions addressing these symptoms in the BDI. Therefore, any assumption about the correlation between BDI and contrast gain must be made very carefully.

In summary, in this study, we reported some specificity with respect to our earlier report of reduced retinal contrast gain as an objective marker of major depression: in the adult patients with ADHD, retinal contrast gain was normal. We replicated the previous finding of a significant correlation between contrast gain and BDI in the healthy subjects, but not in the subjects with ADHD. Thus, the measurement of contrast processing with this stimulus paradigm might be helpful in the differential diagnosis of patients with ADHD and major depression.
